# Learning Experiences Abroad Adding Value in Medical Education (LEAVE): Results From the LEAVE Trainee Experience Survey

**DOI:** 10.7759/cureus.82779

**Published:** 2025-04-22

**Authors:** Dominic Waugh, Andrew Hayburn, Magnus Johnston

**Affiliations:** 1 Trauma and Orthopaedics, NHS Greater Glasgow and Clyde, Glasgow, GBR; 2 General Practice, NHS Education for Scotland West Region, Glasgow, GBR; 3 General Surgery, NHS Lothian, Edinburgh, GBR

**Keywords:** international, junior doctor training, resident, study leave, trainee feedback

## Abstract

Introduction: To facilitate professional development, resident doctors (RDs) in UK training programmes are contractually entitled to take study leave to pursue activity related to progression of training, at the discretion of their Training Programme Director. In Scotland, NHS Education for Scotland (NES) is responsible for managing training delivery and allocating study budgets. In 2024, NES announced a temporary change to study leave policy restricting the use of international study leave for RDs before reversing this decision after pressure from interest groups. Increasingly, RDs in training are reporting high rates of stress, burnout and mental health issues. There are many reasons for this, including reports of financial difficulties, access to study leave and variation in institutional policies. There is relatively little data regarding RD views on funding or the use of international study leave. We are the first to present UK data, highlighting viewpoints on international study leave and funding use by RDs in Scottish training programmes.

Methods: RDs in Scotland's medical specialty training programmes were surveyed using a questionnaire that included both closed and open response questions, designed with collaborative input from authors in diverse medical fields. Descriptive statistical analysis of quantitative data was conducted using Microsoft Forms. Qualitative analysis of free-text responses was conducted with the aid of Google Gemini software.

Results: In total, 348 responses were obtained from a variety of medical specialties and levels of seniority. International study leave had been used by 42% (n = 146) of RDs since commencement of training, with 77% (n = 269) intending to utilise international leave in the future. An overwhelming majority (98%, n = 342) felt concerned about potentially losing the opportunity to take international study leave. RDs reported estimated personal costs to continue training of up to £3,000 (39%, n = 133) and up to £1,000 (47%, n = 159) per annum. Most (80%, n = 278) felt that the study budget in Scotland was not appropriate to support training needs and aspirations. Responses to questions about international study leave on the Likert scale were generally favourable.

Conclusion: This national survey is the largest of its kind in detailing the opinions of RDs regarding international study leave, specifically in terms of perceived value and cost. The data represent a wide range of medical specialisms and grades of seniority.  Overall, the majority of RDs seek to utilise international study leave in the future. The study budget in Scotland does not cover the RD needs and aspirations in the majority of cases, with individuals incurring high personal costs to continue specialty medical training. There is overall support for the use of international study leave and a high level of concern regarding the potential removal of this learning opportunity. Responses highlighted multiple benefits international study leave brings to research and collaboration. Respondents reported reduced levels of stress and increased enthusiasm on return to work. RDs felt international study leave was cost-effective and allowed them to access learning opportunities not available within NHS Scotland. It is of paramount importance to address funding and accessibility concerns regarding international opportunities, ensuring equitable access and preparing future consultants for independent practice.

## Introduction

Study leave for UK resident doctors (RDs) in medical training is defined as contractual 'time inside or outside of the workplace for formal learning that meets the requirements of the curriculum and personalised training objectives' [1]. All RDs within the National Health Service (NHS) are contractually entitled to take study leave, with foundation year 1 doctors allocated 15 days per year and all other training doctors 30 days [2]. Study leave policies in the UK, like many health matters, are devolved and managed separately by national healthcare education bodies. Separate policies exist between Healthcare Education England (HEE) [3], NHS Wales [4], NHS Education for Scotland (NES) [5] and Northern Ireland Medical and Dental Training Agency [6]. Each body adopts different approaches to available funding and access to international study leave.

There is wide regional variation in the provision of the RD study budget across the UK to support study leave activity. While organisations such as NES and NHS Wales set study budgets nationally, RDs from separate regions across England have reported variability in local access to funding. National policies highlight nominated study budgets limited to £600 within Scotland [5] to a maximum approval cap of £1,000 per course in England with 'no upper limit' to the amount that can be claimed, subject to approval processes [7]. An annual Study Leave Report from HEE for the year 2020/2021 indicated a large regional variation in total spending, with some regions reporting an underspend of over £450,000 and others an overspend of nearly £150,000 [8]. The reasons behind this variation are unclear.

Differences in study leave policy [3-6] could potentially disadvantage some RDs depending on the region where they live and work. However, geography is not the only factor influencing access to study leave. A survey of UK Psychiatry Trainees highlighted concerns regarding limited availability and use of study budgets by RDs who are less than full-time (LTFT), international medical graduates (IMGs), living with a disability or from under-represented ethnicities [9]. Study budgets are often further reduced for LTFT RDs, with budgets reduced on a 'pro rata' basis in Scotland [5]. In comparison, Welsh LTFT RDs are allocated study leave time on a 'pro rata' basis, but receive the full year funding allocation [4]. There is potential for this budget disparity to affect female RDs disproportionally: in Scotland, only 18% of LTFT trainees identify as men, although this percentage is increasing [10]. Compared to UK graduates, IMGs have more often reported having no list of mandatory or desirable courses and limited ability to claim travel and accommodation costs, leading to further discrepancies in equitable access to training opportunities [9].

These discrepancies in policy can lead RDs to take on personal costs to continue and complete training. While funding allocated through study budget can offset some of these costs, the variety in funding available across training regions inevitably leads to RDs taking study leave at personal financial deficit. Surgical RDs, training in a 'craft-heavy' specialty with a requirement for multiple practical courses, have reported personal costs ranging from £20,000 to £26,000 throughout training [11]. In response, surgical trainee organisations have begun calling for training to be cost-neutral [12] to reduce the burden to the individual. Study budget caps also affect RDs' training in non-hospital specialties. In Scotland, RDs in General Practice (GP) have 'no automatic entitlement to… funding', with LTFT RDs again reimbursed on a 'pro rata' basis [13]. It is postulated that the GP RD study budgets reflect national spending priorities. While the Darzi report recognised that GPs are one of the most financially efficient parts of the NHS [14], the NHS Long-Term Workforce Plan proposes an increase in the number of fully qualified GPs by only 4% compared to a 49% increase for hospital doctors [15].

This small increase in the workforce for GP RDs could be one of many reasons contributing to high levels of stress and burnout in recent surveys [16]. Other large surveys of RDs have reported similarly high levels of stress, mental health concerns and low morale [17,18]. The Association of Surgeons in Training has suggested that one such method to aid work-life balance would be for automatic approval of 'annual or study leave with >6 weeks notice…regardless of assigned duties' [19]. Accordingly, the use of RD leave and the associated effects on stress levels are receiving increased attention in training discussions. There is little direct evidence describing how leave policy influences RD levels of stress and burnout, with much of the literature focussing on parental leave policies in American Residents. Inconsistent leave policies across different specialties and institutions have been shown to lead to higher levels of stress and dissatisfaction in American Surgical Residents working in less supportive environments [20]. Other surveys have revealed that American Medical Residents are strongly considering leaving residency due to inadequate parental leave support [21].

Further inconsistency in UK study leave policy was highlighted in 2024 when NES updated national study leave policy via an email to RDs, highlighting that 'in-person attendance and associated travel, accommodation and subsistence costs, will not be supported for overseas events'. While this decision was later reversed, this move attracted significant criticism from professional representation bodies [22].

Surveys have highlighted a desire for RDs to attend face-to-face meetings, suggesting they would be more likely to do so if they had access to study leave [23]. While some RDs choose to pursue international courses, the precise number of those doing so remains unclear. Although the COVID-19 pandemic severely restricted international academic gatherings, research has since reaffirmed their crucial role in academic and societal advancement [24]. There is evidence to support RDs attending international learning events, with literature suggesting that international experiences are beneficial for both the individual and the wider healthcare system. IMGs comprise a large proportion of licensed doctors in the UK, with around a third graduating abroad, highlighting the importance of international experience in developing and maintaining the UK healthcare sector [25]. Clinicians undertaking international work have reported increased clinical skills, involvement in research and service development activity upon return to the UK [26]. Studies have shown that extended research stays abroad enhance scientific progress and productivity, as scientists with longer international experiences are more effective at transferring knowledge both domestically and internationally [27].

Existing research describing the RD experience of international study leave and the use of study budget within NHS Scotland is limited. The authors noted high levels of anecdotal concern among peers regarding proposed changes to the international study leave policy in NHS Scotland. In response, we aimed to analyse perceived benefits, costs and potential impacts on training by documenting RD attitudes towards international study leave. We also explored RD perceptions of the potential effects of reducing access to this learning opportunity. Additionally, we sought to quantify RD usage and intentions regarding international study leave to develop areas for future research and policy development. To our knowledge, this is the largest survey evaluating RD views and future intentions regarding the use of international study leave.

## Materials and methods

The Learning Experiences Abroad adding Value in medical Education (LEAVE) survey questions were designed by the authors to evaluate a range of RD opinions related to International Study Leave and collate perspectives related to training opportunities outside of the UK. Questions were designed after discussions with various working parties about the issues faced by RDs and the advantages and disadvantages posed by potential changes to policy, to generate an 'expert' consensus. After the design, the authors invited colleagues to review the content for structure and clarity before its general release. The survey incorporated various question types, including closed (with participants required to select from pre-defined options) and open-ended free-text responses. Additional questions utilised Likert scales ranging from 1 to 5 with descriptors ranging from 'completely disagree', 'somewhat disagree', 'neutral', 'somewhat agree', and 'completely agree'. The use of balanced question phrasing minimised response bias. The survey was published in Microsoft Office Forms (Microsoft Corp., Redmond, WA), a versatile web-based tool integrated within the Microsoft Office Suite (Microsoft Corp.), facilitating the creation of online surveys for data collection.

The target population included all RDs within specialty training programmes in Scotland. While RDs were encouraged to contribute insights via the LEAVE survey (see the Appendix), no incentive or prize was offered. All RDs in Scotland were eligible to complete the survey, confirmed by answering 'Yes' when asked on the survey if the respondent was a 'Scottish RD with a national training number'. Respondents from non-training posts were excluded as local employment contracts are negotiated directly with the employer and not subject to the NES study leave policies. Official statistics for the number of RDs (medical trainees) in Scotland as of 2024 highlight 'whole time equivalent' numbers of 6,570, which includes multiple LTFT trainee posts grouped together and, therefore, more potential respondents. Additionally, these numbers also include Foundation Doctor posts, which were not eligible for inclusion. Therefore, it is challenging to specify the potential number of eligible respondents.

The survey was disseminated to all Scottish Specialty Training Programme Directors' email addresses (approximately 170), which were publicly accessible through the NHS Scotland online domain, with a request for regional circulation amongst trainees. A limited number of email addresses were invalid. Further distribution was achieved through targeted social media and direct email communication via Microsoft Office Outlook (Microsoft Corp.). The survey was open for completion over a six-week period, from 1 October 2024 to 10 November 2024. This limited response period allowed for an efficient 'snapshot' of opinion at a time of rapid policy change within NES. To maintain data integrity, additional security protocols were activated on Microsoft Office Forms to allow only respondents with a verified NHS Scotland email address to respond. Each email address was only able to respond to the LEAVE survey once to avoid duplicate responses. All responses were suitably anonymised. Data were collected digitally and stored securely in line with the General Data Protection Regulations via NHS Outlook. Responses were summarised in graphical and tabular format via Microsoft Office Forms. The authors then analysed the results to identify trends and differences.

Qualitative analysis of anonymised free-text comments was conducted with assistance from Google Gemini software (Google, Mountain View, CA), a generative AI model designed to understand trends in human language when prompted to undertake thematic analysis. Prompts given to Gemini reflected requests for thematic analysis of key themes. These were identified based on frequency and contextual relevance, followed by manual review by each author to determine trends and minimise specialty-specific biases. The results are reported aggregates, ensuring that no individual participant can be identified.

No institutional or ethical review board approval was sought for this survey, as the authors conducted this survey independently. A participation statement was provided, highlighting that responses may be used for publication and presentation.

## Results

In total, 348 responses were obtained: all of these were from RDs in Scottish Medical Training. All results are expressed as whole numbers and percentages to the nearest whole decimal.

**Figure 1:**
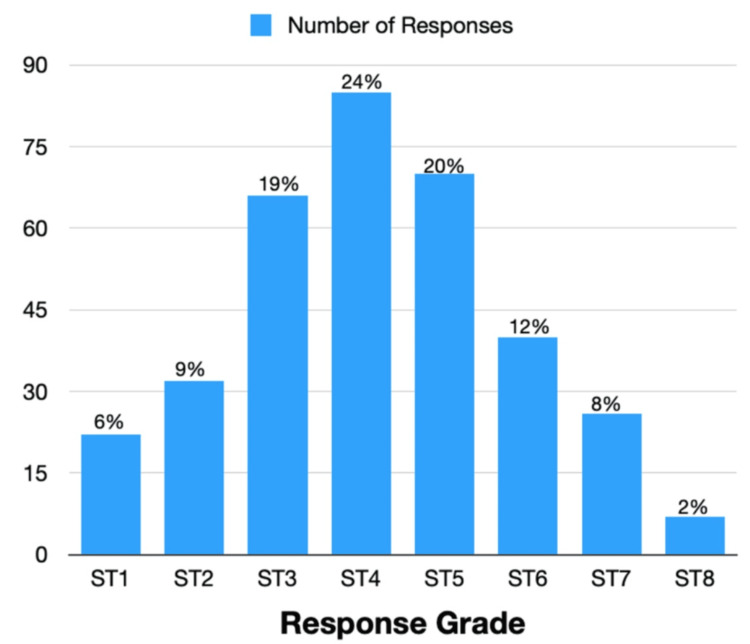
Number of responses by trainee grade

Responses were obtained from various levels of RD seniority: ST1 (6%, n = 22), ST2 (9%, n = 32), ST3 (19%, n = 66), ST4 (24%, n = 85), ST5 (20%, n = 70), ST6 (12%, n = 40), ST7 (8%, n = 26) and ST8 (2%, n = 7). Responses by grade are expressed as the number of respondents or percentages in Figure 1.* *Of note, several training programmes end at ST3 (GP) or at ST5 (Radiology), while others (Surgery) finish at ST8.

**Figure 2:**
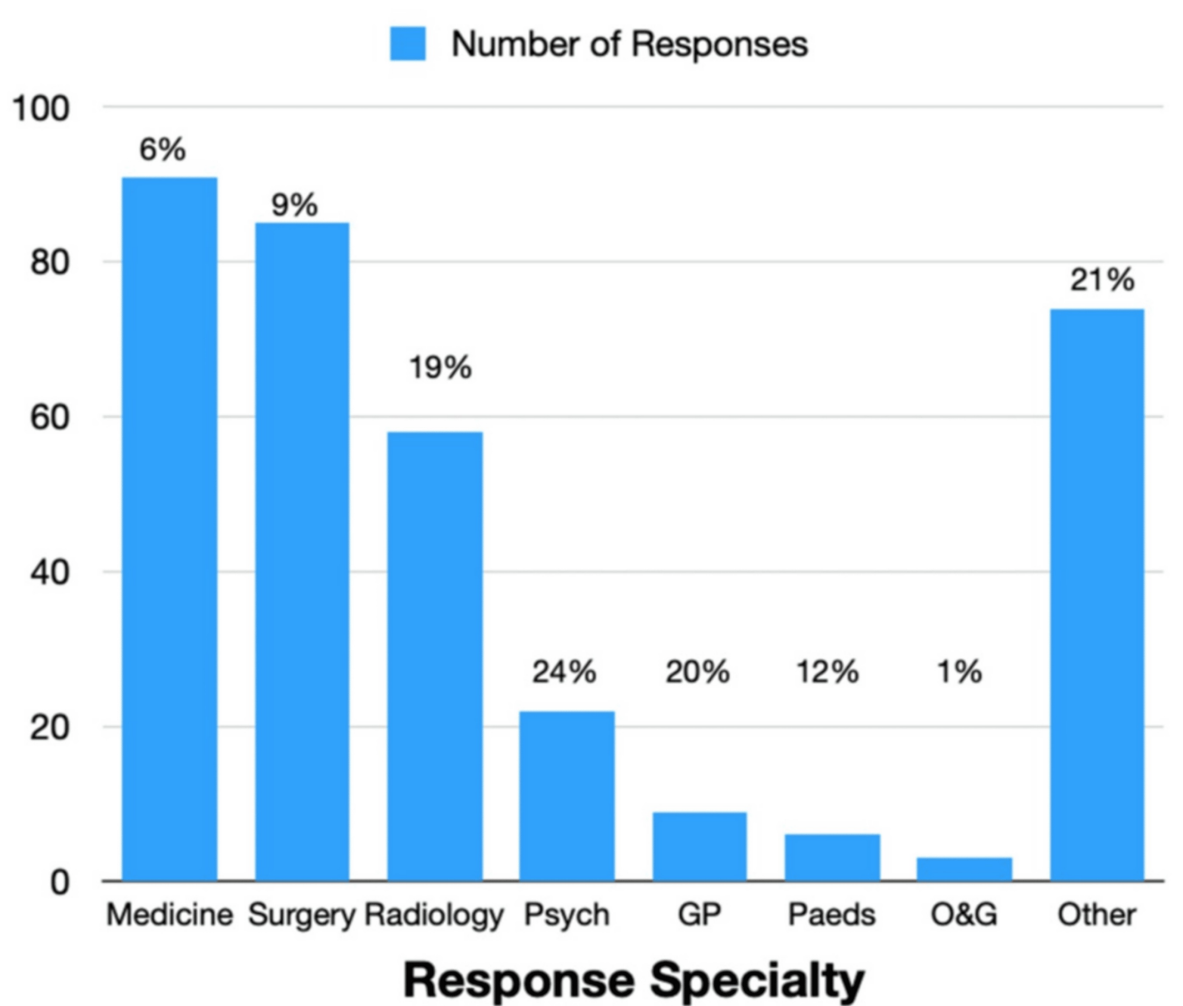
Number of responses by trainee specialty GP: General Practice; O&G: obstetrics and gynaecology; Psych: psychiatry; Paeds: paediatrics

A wide variety of specialties responded, including: Medicine/Medical Subspecialty (26%, n = 91), Surgery/Surgical Specialties (24%, n = 85), Radiology (16%, n = 58), Psychiatry (6%, n = 22), GP (2%, n = 9), Paediatrics (2%, n = 6), Obstetrics and Gynaecology (1%, n = 3) or Other (21% n = 74). Responses by specialty are expressed as the number of respondents or percentages in Figure 2.

**Figure 3:**
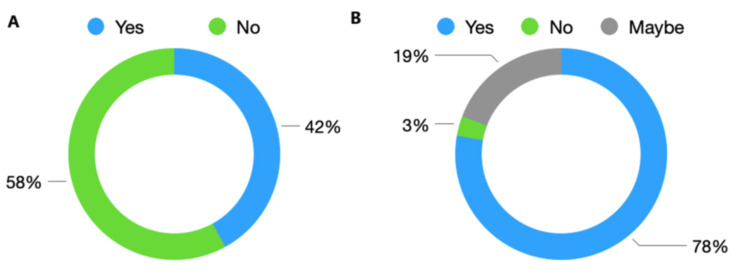
Trainee responses regarding (A) previous and (B) future intentions to use international study leave

In total, 42% (n = 146) reported having used international study leave since starting training, while 58% (n = 144) had not used international study leave. When asked whether they intended to take international study leave in the future, 77% (n = 269) stated 'Yes' to intention to take this in the future, with 19% (n = 67) indicating 'Maybe'. A minority of 3% (n = 12) stated they did not intend to take international study leave in the future. These responses are summarised in Figure 3.

**Figure 4:**
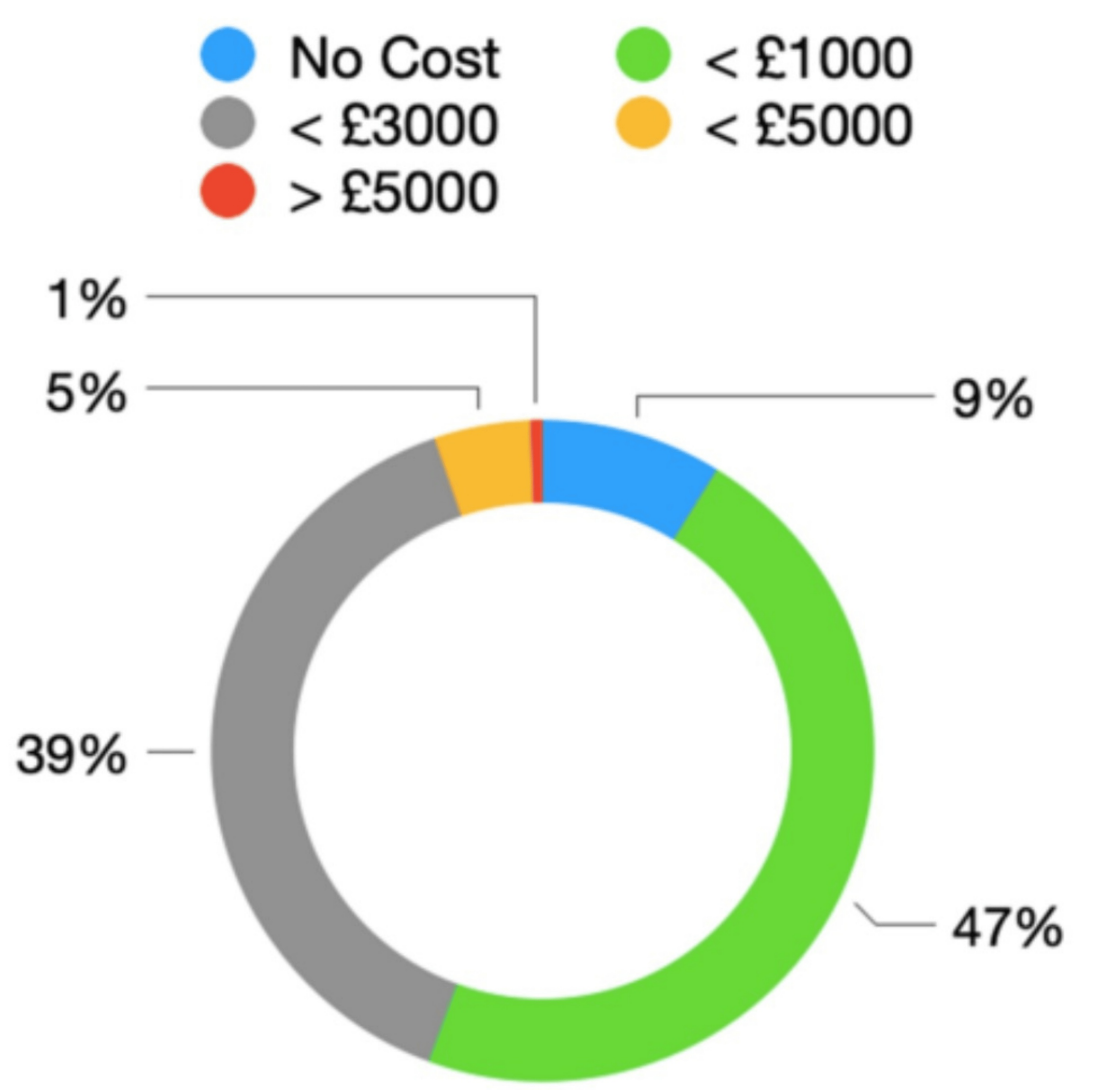
Estimated personal costs to resident doctors to continue training (last 12 months)

RDs were asked to estimate their personal training costs over the last 12 months. Responses ranged from no personal cost for 9% (n = 30), up to £1,000 for 47% (n = 159), up to £3,000 for 39% (n = 133) and up to £5,000 for 5% (n = 16). Two participants reported a personal expense of more than £5,000 (1%, n = 2). This is summarised in Figure 4*.*

**Figure 5:**
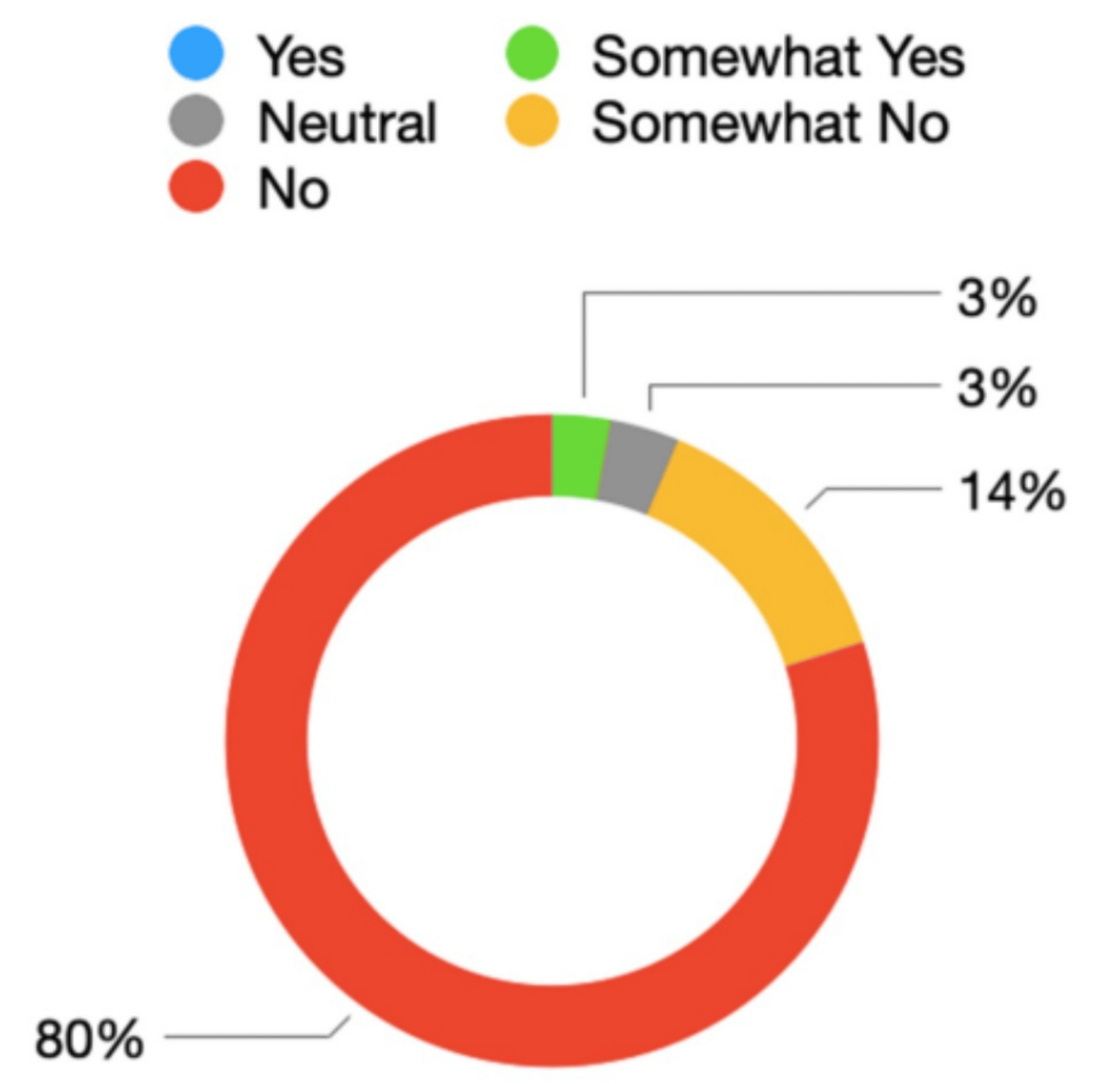
Trainee opinions on suitability of study budget to support needs and aspirations

When asked if the study budget covered all mandatory training costs for the year, 91% (n = 318) of RDs stated that the study budget did not cover all training costs, while 9% (n = 30) stated that the study budget covered all training costs. Respondents were asked whether they felt the current £600 annual training budget in Scotland is appropriate to support training needs and aspirations. In total, 80% (n = 278) reported 'no' and 14% (n = 48) reported 'somewhat no'. The study budget was felt to be appropriate by 3% (n = 10) of respondents. This is summarised ​​​​​​​in Figure 5​​​​​​​*.*

**Figure 6:**
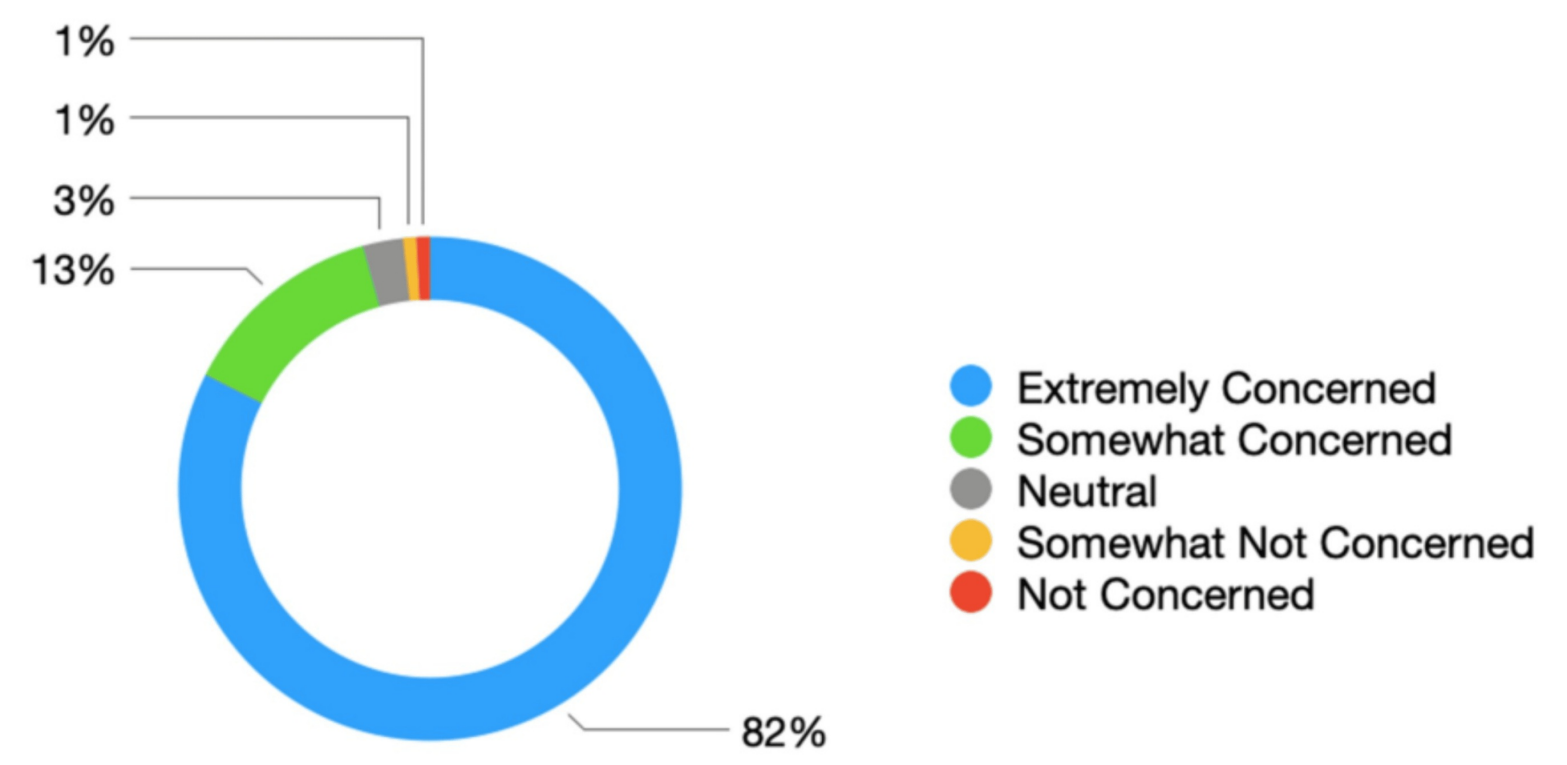
Trainee concern around potential removal of international study leave

Participants were asked about their perceptions of NES's previously indicated change to (removal of) the budget for study leave for overseas events. An overwhelming majority of 82% (n = 287) reported feeling 'Extremely Concerned' regarding the potential removal of study leave, with 13% (n = 46) reporting being 'Somewhat Concerned' by the policy change. A smaller 3% (n = 9) reported being neutral to any changes, with a minority (n = 9) reporting feeling 'Somewhat Not Concerned' or 'Not Concerned'. This is summarised in ​​​​​​​Figure 6​​​​​​*.*

In relation to international study leave for overseas events specifically, RDs were asked to agree or disagree with a number of statements using a Likert scale of 1-5 (ranging from completely disagree to completely agree). Responses were categorised as either agreeable (4-5), neutral (3) or disagreeable (1-2). Questions were phrased in such a way as to describe both possible benefits and possible drawbacks to overseas events to minimise response bias. This is summarised in Table 1 and elaborated on further in the Discussion section.

**Table 1:** Likert scale responses to study leave statements CCT: Certificate of Completion of Training

Statement	Completely disagree	Somewhat disagree	Neutral	Somewhat agree	Completely agree
I enjoy collaboration with international peers	<1%	<1%	3.5%	15%	81%
My work/research is better with international collaboration	<1%	2%	6%	16%	75%
I am less stressed about my job when I return from international study leave	2%	3%	27%	20%	48%
I learn less on international study leave	68%	14%	13%	2%	3%
I return to work more enthusiastic about my job after an international study leave	1%	<1%	12%	18%	69%
International study leave reduces stress/burnout	<1%	1%	16%	23%	60%
International study leave is not value for the money	66%	18%	11%	4%	1%
I can undertake similar/the same international courses cheaper in the UK	57%	28%	12%	3%	<1%
I can undertake a funded international learning opportunity (industry funding, fellowship, scholarship, etc.)	11%	21%	26%	20%	22%
I can undertake an international learning opportunity not available in the UK	5%	5%	12%	27%	51%
Removing international study leave will reduce the quality of medical training in Scotland	<1%	2%	3%	11%	84%
International study leave can help me achieve CCT	2%	3%	14%	23%	58%
International study leave can help me secure a consultant job/posting after CCT	2%	1%	11%	18%	68%
Removing international study leave will have no effect on the recruitment and retention of trainees in Scotland	72%	15%	6%	3%	4%

A qualitative analysis of free-text responses was conducted with the aid of the Google Gemini software as described in the Methods section*.* Several key themes were highlighted, including potential for professional development, personal advantage and wider advantages to NHS Scotland. Concerns were raised regarding the disadvantage to RDs in limiting study budget and recommendations for future policy. Free-text responses are summarised in Table 2 and elaborated on in the Discussion section.

**Table 2:** Qualitative analysis of free-text comments RDs: resident doctors; CCT: Certificate of Completion of Training; LTFT: less-than-full-time; IMGs: international medical graduates

Identified theme	Free-text analysis
Professional development and career advancement	RDs highlighted several advantages felt to be gained by the use of international study leave. Respondents felt that by networking with international colleagues and developing relationships, there was opportunity to foster collaborations leading to joint research projects with international peers. Attending international learning opportunities has the capacity to improve patient care, facilitating access to specialised courses and learning from leading international experts. The ability to attend unique training opportunities not provided within NHS Scotland was highlighted, particularly among trainees in highly small specialist subdisciplines and in surgical trainees. RDs felt able to learn from the latest advances and apply this to benefit patients in NHS Scotland by using international study leave. There is the added advantage of facilitating understanding of diverse healthcare systems, gaining insight in to different healthcare models and delivery of care
Personal advantages, stress and burnout	RDs reported increased motivation and satisfaction with regard to their work when returning from international study leave. Experiencing new health care cultures and perspectives refreshes RD perspective and motivates them on return to work. By breaking from routine and gaining fresh inspiration, responders report reduced burnout. RDs are able to gaining experience and recognition to improve CVs and secure competitive positions. This helps them develop to provide the “best” possible care for Scottish patients. RDs return from international study leave with enhanced confidence and self-esteem, gained by presenting research and participating in international forums
Advantages to the Scottish healthcare system	Respondents highlighted the ability to attain learning not available within the UK and bring this back to Scotland to develop and deliver healthcare, which is world leading. It is also highlighted that this learning can be more efficient in the International setting, such as increased operative numbers for surgical trainees at major international centres of excellence or attendance at specialist medical clinics in areas with high disease burden to increase confidence in managing complexity. Trainees in highly specialist disciplines highlighted the need for international collaboration to advance care for patients in Scotland. It is highlighted that Scotland has a significant shortfall in appointed consultants compared to other UK regions. By supporting international study leave, there is potential to attract talent and reduce workforce shortages. There is potential to strengthen the reputation of Scottish Healthcare and contribute to global medical advancements, further enhancing recruitment and retention. While there are immediate costs associated with international study leave, some courses can be less financially costly internationally than domestically leading to upskilling of the workforce
Disadvantages of limiting study budgets and international leave	Trainees believe they may be at a disadvantage, with fewer opportunities for collaboration and research, compared to other parts of the UK and international colleagues if international study leave is not funded. Reduced research opportunity has the potential to not only damage patients through nonparticipation in advancing healthcare but also damage the professional development and reputation of doctors in Scotland. The current study budget is widely viewed by trainees as insufficient to cover the costs associated with medical training in Scotland. Some trainees report bearing significant personal financial costs. The current study budget constraints do not cover even “mandatory” training costs for many programmes, let alone “aspirational” courses to improve patient care. Trainees are concerned that the delivery of training does not even cover the “bare minimum” and that limiting opportunities will drive down the quality of care in Scotland. Trainees perceived limitations on international study leave as diminishing the recognition of their professional contributions. There is significant concern that decisions are being made without consultation by individuals with limited knowledge or understanding of how this will affect them. Trainees believe fewer funded training opportunities would limit access to specialised courses, which may ultimately lead to poorer patient outcomes. There are concerns affecting trainees disproportionately. Part-time trainees may be particularly disadvantaged by the reduced study budget. Trainees from lower socioeconomic backgrounds may be less able to personally fund international travel and conference fees. Aspirational development is less likely to be possible. This risks a “two-tier” system of training across Scotland
Trainee recommendations for future policy makers	Increases in the available study budget are viewed as necessary to cover the costs of training and attaining CCT. Trainees desire a streamlined approval process for the use of the study budget and relative autonomy in spending this, acknowledging the need for appropriate oversight from their own Training Programme Director. Trainees value international study leave for their professional development as the future of the medical profession. Ensure that all trainees, regardless of their background or professional circumstances, have equal access to national and international training opportunities and funding to attend these. Particular attention should be paid to LTFT trainees and IMGs, as well as support specialised for each training program. Address environmental concerns by exploring alternative approaches, such as virtual attendance, carbon offsetting or more environmentally friendly forms of transport to reduce the environmental impact of international travel

## Discussion

Overall trends in RD responses

This national survey is the largest project examining the opinion of RDs toward international study leave regarding perceived value and financial cost. Data represent a wide range of medical specialisms and grades of seniority. There is an overwhelming desire among RDs working in NHS Scotland to take international study leave, with 77% (n = 269) intending to utilise international study leave in the future. There is great concern regarding the impact a loss of international study leave would have on training: 95% (n = 333) of respondents felt 'Extremely Concerned' or 'Somewhat Concerned', suggesting that this topic is of importance to RDs. Participants report that NHS Scotland study budget is not adequate or appropriate to support RDs in their development in over 94% (n = 326) of cases, with respondents taking on an average personal cost of between up to £1,000 (47%, n = 159) and up to £3,000 annually (39%, n = 133). In a limited number of cases, RDs reported spending over £5,000 (5%, n = 16). While this level of financial burden for Scottish RDs may be explained by the difference in study leave policy between devolved nations [3-6], the non-financial costs to personal lives [19] in pursuing Scottish Medical Training should also be considered.

Responders were asked to agree or disagree with a number of statements regarding international study leave. RDs agreed they enjoyed collaboration with their international peers (96% agreement) and felt work and research were better with international collaboration (92% agreement). RDs reported that they were less stressed on return from international study leave (70% agreement), returned to work more enthusiastic (90% agreement) and experienced reduced levels of stress/burnout (85% agreement). While these self-reported trends in stress reduction associated with international study leave are of interest, causality cannot be established due to the survey design. NHS Employers have publicly issued guidance on reducing stress and burnout in trainees [28]: it is plausible that international study leave could play a role in helping to address this issue. However, further research around this topic is required.

There was agreement that international study leave could help achieve curriculum requirements for completion of training (83% agreement) and would help secure a consultant post (78% agreement). This is in keeping with previous work on the social and academic benefits of international academic conferences [24]. RDs disagreed that they learnt less effectively on international study leave (84% disagreement). Opportunities not present in the UK were felt to be available through international study leave (78% agreement), and a limited number believed they could access additional funding to access an international learning opportunity (42% agreement).

RDs did not believe that international study leave represented poor value for money (87% disagree). Further, qualitative analysis comments highlighted that international study leave could be less expensive than attending similar courses within the UK. With the highlighted cost implications of pursuing medical training in Scotland from this survey and limitations of the current study leave policy in Scotland [5], it is interesting that international study leave could potentially help reduce costs while meeting training curriculum requirements.

Participants disagreed that removing international study leave would have no effect on the recruitment and retention of RD in Scotland (87% disagreement). While medical training post numbers in Scotland have demonstrated a slight improvement in fill rates in 2024, trade unions have highlighted that consultant vacancies are believed to be more than double the Scottish Government’s official figures, with a recruitment and retention crisis across Scotland [29]. The responses to this survey suggest that study leave could be an important area for policy developers to consider in relation to the recruitment and retention of medical staff.

Qualitative analysis

A qualitative analysis of free-text responses was conducted with the aid of the Google Gemini software as described in the Methods section. Several key themes were highlighted, including potential for professional development, personal advantage and wider advantages to the Scottish healthcare system. Concerns were raised regarding the disadvantage to RDs in limiting study budget and recommendations for future policy. Key themes are noted in the Results section. It is of note that RDs are increasingly choosing to pursue international fellowships after completion of training [30]; respondents reported that international study leave can help develop key relationships to develop fellowship opportunities. By reducing access to international study leave, Scottish RDs could be disadvantaged compared to other UK regions. A selection of LEAVE survey comments utilised as part ofthe thematic analysis is included in Table 3.

**Table 3:** Selection of free-text comments from LEAVE survey LEAVE: Learning Experiences Abroad adding Value in medical Education; CCT: Certificate of Completion of Training; LTFT: less-than-full-time; NES: NHS Education for Scotland

Identified theme	Free-text comment
Professional development and career advancement comments	‘I will have to… give up being able to achieve my career goals’. ‘I am a trainee in a highly specialist area of practice. There are essential courses needed for my progress organised exclusively by international organisations. Adequate knowledge cannot be achieved through other national meetings’. ‘Unacceptable decision. International courses are extremely helpful for CCT requirement and progression’. ‘I recently presented my PhD work at an international conference, providing important opportunities for professional development’. ‘I do have an expectation I have to go abroad at some point’. ‘Without points gained from these (international) experiences, I would not have been listed for interviews in my specialty training application’.
Demographic comments	‘The limited study budget makes access to professional development inequitable for doctors from less affluent backgrounds, LTFT or protected characteristics’. ‘Experience outside the UK allows clinicians to appreciate differences in pathology and its management that are often under-represented in the UK. Limited knowledge can negatively impact BAME patients in the UK’. ‘As an LTFT trainee, I have been very concerned. The majority of trainees (in my specialty) are women and most are LTFT. We take a big pay cut…. Attending important courses becomes impossible… I have had to stomach the personal costs of care for my toddler… My husband can’t take annual leave…’
Wider healthcare system comments	‘I have learn a lot more and brought more back to my practice and department from being abroad’. ‘I learned surgical techniques not done in Scotland on a travelling fellowship, had access to cadaver labs to practice which juniors can’t access in UK’. ‘How can we provide world-class training without the opportunity for international leave?’ ‘I am an SpR… Many of our conditions are ultra rare. It is only through working with international collaborators we can learn more about these conditions and give high quality care to patients… I am working with a group in America and with another group in the Netherlands… Many of our conferences are run by the European Society’. ‘I am learning an international research group including members of the World Health Organisation… this is simply not possible in a virtual setting’. ‘This decision will immediately and directly reduce the quality of care patients receive in Scotland’. ‘This decision will lead to Scottish consultants becoming more insular and fall behind other UK nations and overseas in terms of research, innovation, capability and patient outcomes’. ‘Completely isolating trainees who need exposure to the international community… Unbelievable they would put though this change without consultation’
Cost comments	‘I have managed to secure funding for international courses… This has given me increased experience and skills with no cost to the NHS except the days I am not at work’. ‘The current study budget doesn’t cover all costs… It is ridiculous that I have to pay for work related travel, leading me to struggle financially’. ‘The current £600 was completely insufficient to cover basically any conferences the UK never mind international’. ‘Trainees have always been out of pocket’
Recruitment and retention comments	‘It will affect training adversely and will disadvantage Scottish Trainees compared to other trainees in the UK’. ‘As a trainee, we will fall behind our peers in England…Had this been announced last year, I would have reconsidered my decision to return Scotland for specialty training’. ‘This is short sighted from NES, training in Scotland is undersubscribed across all areas, taking away benefits of training will only lose trainees’. ‘It is short-sighted, demeaning and insulting to remove this support from Scottish trainees’. ‘Would greatly affect retention of doctors in Scotland - an unfair step comparing to colleagues in England’

Policy recommendations

The LEAVE survey demonstrates overall consensus from RDs on the benefits of international study leave as part of specialty training programmes, with multiple benefits to the individual and wider healthcare system. There is significant concern among RDs that removing international study leave could impact the training of doctors in Scotland. These insights provide additional specialty-specific concerns that have the potential to inform future medical education policies within NES. Given the findings within the limitations of this survey, the authors would recommend that substantial policy changes limiting access to international study leave be suspended until there is extensive consultation with relevant trainee associations and stakeholders. It is felt that the current study budget is inadequate for RDs in Scottish Training Programmes due to the high levels of personal costs experienced by trainees and potential disadvantages experienced by those in Scotland compared to other devolved nations. As a priority, authors would recommend efforts to increase available study budgets to RDs in Scotland annually to match those training in other UK regions. This should include a study budget to cover the cost of mandatory requirements and adequately support LTFT trainees.

Limitations

The response rate to this survey (n = 348) represents a small proportion of the total RDs in training overseen by NES. While efforts were made to minimise selection bias by encouraging responses from as many RDs as possible, it is possible that there is less motivation for participant involvement in specialties less affected by change to study leave policy and affected by budgetary constraints. As such, there is a risk that those with strong opinions were over-represented.

The use of Google Gemini software to aid thematic analysis shows a potential lack of contextual understanding, with reliance on algorithmic interpretations of free-text comments. The effects of this were minimised with manual author review as described in the Methods section.

While the authors deliberately chose to avoid collecting demographic data to aid anonymity in responses, we are therefore limited in our ability to explore differential impacts across subgroups (gender, ethnicity, LTFT status and IMG status). Additionally, we felt that collecting these data was inappropriate given the lack of formal ethical approval processes and potential sensitivity of this topic.

After a request for the training programme director's circulation, one author notes that they were contacted separately by RDs who informed them that their training programme director had specifically requested that they not fill in this survey. This further highlights this topic as an area of potential controversy.

While the authors feel that responses are representative of the majority of RDs in Scotland and are likely generalisable across the UK, further research on specialty-specific differences in attitude toward international study leave and budget use could aid in the development of policies targeting specific support measures for RDs in training.

## Conclusions

The LEAVE survey of RDs in NHS Scotland shows relative consensus on the value of international study leave and available study budget. RDs believe international experiences offer unique learning opportunities, enhance research and collaboration, contribute to curriculum completion and consultant applications, and potentially reduce stress and burnout. While trainees recognise the benefits of professional development, networking and career competitiveness, there are concerns about the equity of funding in Scotland, with many incurring high personal costs. There is a significant lack of funded study leave available through NES. Our results suggest that increased support, both domestically and internationally, could improve the recruitment and retention of RDs in NHS Scotland. Trainees believe enhanced study budgets and international opportunities would benefit their development and the wider healthcare system. We hope these findings will lead to progressive advancements in decision-making, policy development and workforce planning.

## Appendices

Copy of the LEAVE survey

**Table 4:** Copy of the LEAVE survey LEAVE: Learning Experiences Abroad adding Value in medical Education

Question	Available responses							
Are you a Scottish medical trainee with a national training number?	Yes	No	-	-	-	-	-	-
What is your declared specialty?	General practice	Medical specialty practice (Cardio, Resp, Gastro, etc.)	Paediatrics	Psychiatry	Radiology	Obstetrics and Gynaecology	Surgical specialty (General, T&O, Vascular, etc.)	Other
What is your training grade?	ST1	ST2	ST3	ST4	ST5	ST6	ST7	ST8
Have you used international study leave since starting training?	Yes	No	-	-	-	-	-	-
Do you intend to take international study leave in future?	Yes	Maybe	No	-	-	-	-	-
Does your study budget cover all your mandatory training costs for the year?	Yes	No	-	-	-	-	-	-
If your study budget does not cover all training costs in your specialty, what are your estimated personal costs to continue training in the last 12 months?	No personal costs	Up to £1,000	Up to £3,000	Up to £5,000	More than £5,000	-	-	-
Do you feel the current £600 annual training budget is appropriate to support your training needs and aspirations in your specialty?	No	Somewhat no	Neutral	Somewhat yes	Yes	-	-	-
Are you concerned about potential changes to study leave restricting access to international study leave?	Not concerned	Somewhat not concerned	Neutral	Somewhat concerned	Concerned	-	-	-
With regard to international study leave, how strongly do you agree or disagree with the following? All questions are noted as full responses in Table 2	Completely agree	Somewhat agree	Neutral	Somewhat disagree	Completely disagree	-	-	-
Do you have any positive comments about international study leave? What benefits has it brought you?	Free-text response							
Do you have any negative comments about international study leave? What negatives are there in providing this opportunity to trainees in your region?	Free-text response							
